# Rift Valley fever during the COVID‐19 pandemic in Africa: A double burden for Africa's healthcare system

**DOI:** 10.1002/hsr2.468

**Published:** 2022-01-06

**Authors:** Olivier Uwishema, Elie Chalhoub, Tania Torbati, Success Chekwube David, Carlo Khoury, Lucas Loiola Ponte Albuquerque Ribeiro, Yves Nasrallah, Bezawit Kassahun Bekele, Helen Onyeaka

**Affiliations:** ^1^ Oli Health Magazine Organization Research and Education Kigali Rwanda; ^2^ Clinton Global Initiative University Research and Education New York New York USA; ^3^ Faculty of Medicine Karadeniz Technical University Trabzon Turkey; ^4^ Faculty of Medicine University of Saint Joseph of Beirut Beirut Lebanon; ^5^ Department of Osteopathic Medicine of the Pacific Western University of Health Sciences Pomona California USA; ^6^ Faculty of pharmaceutical Sciences University of Nigeria Enugu Nigeria; ^7^ School of Medicine University of Fortaleza Fortaleza Brazil; ^8^ School of Medicine & Medical Sciences Holy Spirit University of Kaslik Beirut Lebanon; ^9^ Addis Ababa University College of Health Science, School of Medicine Addis Ababa Ethiopia; ^10^ School of Chemical Engineering University of Birmingham Birmingham UK

**Keywords:** Africa, burden, COVID‐19, Rift Valley fever

## Abstract

A new rising incidence of Rift Valley fever (RVF) among livestock and humans in the African continent during the COVID‐19 pandemic has become of increasing concern. We analyzed the different ways COVID‐19 has contributed to the increase in RVF cases and how it has impacted the interventions allocated to the disease by comparing it with the status of the disease before the pandemic. There is enough evidence to conclude that the COVID‐19 pandemic has impacted the efforts being taken to prevent outbreaks of RVF. Therefore, with no definitive treatment in place and inadequate preventive measures and disease control, RVF may potentially lead to a future epidemic unless addressed urgently.

## 1 INTRODUCTION

Less than a century ago, a new virus causing fever was discovered in the Rift Valley region of Kenya, thereby naming the new organism the Rift Valley fever virus (RVFV).^1^ Although more than 90 years have passed, the virus' real impact and treatment are still misunderstood.^1^ The situation led the World Health Organization (WHO) and many African nations to prioritize the pathogen with respect to assigning it more urgent research and development.^2^, ^3^

**FIGURE 1:**
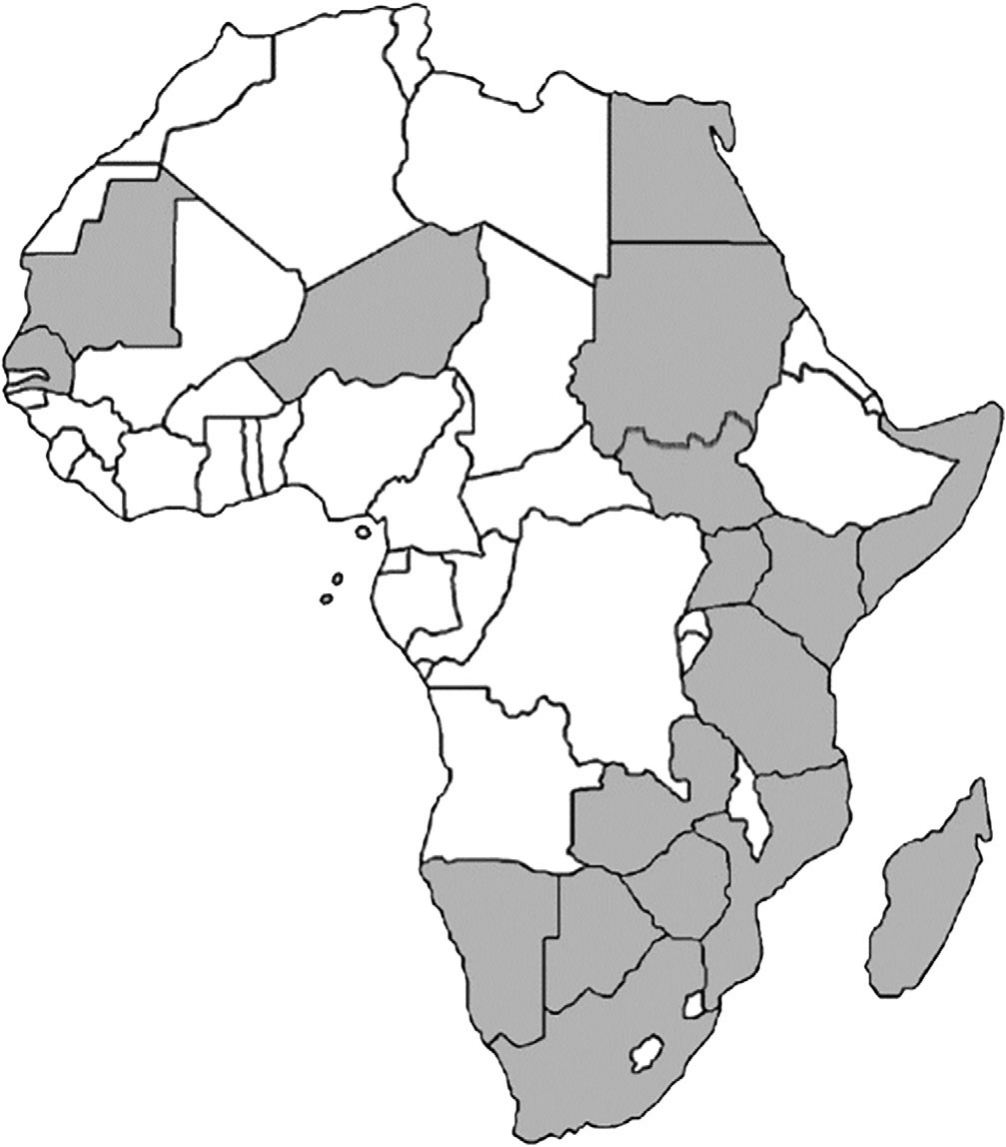
African countries with endemic and outbreaks of RVF disease. List of countries affected in grey: Egypt, Gambia, Kenya, Madagascar, Mauritania, Mozambique, Namibia, Senegal, South Africa, South Sudan, Sudan, Tanzania, Uganda, Zambia, Zimbabwe

RVF is a disease that attacks mainly domesticated ruminants and sometimes humans in sub‐Saharan Africa, particularly the East and South regions, Egypt, Saudi Arabia, and Yemen (Figure 1).^4^ Since its detection, different outbreaks have been reported, with the latest one taking place in May 2021 in Madagascar (http://www.fao.org/emergencies/fao-in-action/stories/stories-detail/en/c/1419441/).

During only the first 6 months of 2021, Africa witnessed 5130 cases of RVF, 1500 deaths, and 27 900 susceptible cases (https://wahis.oie.int/#/dashboards/qd-dashboard)

In addition to the major economic and social impacts these outbreaks have, RVF's effect on human lives is notably concerning. For instance, RVF causes the loss of nearly all livestock pregnancies and young animals, and the mortality rate in adult animals is around 20%.^4^ Furthermore, even though most human infections go unnoticed, about 10% of people develop severe illnesses such as ocular disease, encephalitis, and hemorrhage, while also leaving 2% of infected individuals with vision loss or neurological deficits and subsequent death in another 1%.^1^, ^4^ Also, a new research study found that RVFV can lead to placental dysfunction,^5^ and this claim was endorsed by the WHO.^1^

With global travel and climate change, the above‐mentioned facts become more concerning as mosquito vectors may spread to different regions of the world and infect other areas currently deemed untouched.^4^, ^6^ To prevent a new pandemic risk, this study aims to shed more light on RVF by exposing its current burden and efforts to fight it amidst COVID‐19.

## 2 RIFT VALLEY FEVER BEFORE THE COVID‐19 PANDEMIC IN AFRICA

For 70 years, the virus that causes the renowned RVF has been responsible for major outbreaks targeting mostly lifestock.^1^ Specifically, between 1950 and 1951, Kenya suffered vast losses in livestock while facing the first ever major outbreak of RVF. It was not until the South African outbreak in 1974 that the first ever human death due to RVF was recorded.^7^ Consequently, it became clear that RVF posed an economic threat to communities that rely on the meat, milk, and trade of affected livestock.^8^

The most suitable approach to prevent the critical dangers of RVF is via immunizing animals with one of a variety of vaccinations.^1^ Because multiple doses of immunization are required, inactivated or killed vaccines are not appropriate for regular animal field vaccination. The Smithburn vaccine, a modified live vaccine, is Africa's oldest and most used vaccine for preventing RVF. This vaccine conveniently requires only one dose; however, it has been linked to birth abnormalities and demonstrated low effectiveness.^4^

Several potential vaccinations are currently being researched and tested. In laboratory studies of domesticated animals, the live‐attenuated vaccine MP‐12 has shown promise, but further investigation is needed before the vaccine can be deployed in the field. Of note, South Africa has approved and started using the live‐attenuated Clone 13 vaccine. Also, alternative vaccines based on molecular recombinant constructions are now being developed.^4^ Although frequent outbreaks still exist in the 21st century, vaccination has played a major role in reducing the severity of these epidemics.^1^

## 3 BURDEN AND CURRENT STATUS OF RIFT VALLEY FEVER IN AFRICA DURING THE COVID‐19 PANDEMIC

A new outbreak of RVF has recently emerged in Africa, with 109 confirmed and suspected cases in Madagascar according to the WHO^9^ and 4 deaths in Uganda.^10^ Unfortunately, this outbreak appeared during the SARS‐CoV‐2 global pandemic, which made the situation worse. RVFV and SARS‐coV‐2 both share some common clinical features: for example, in their early course, the two viruses cause flu‐like, febrile diseases that may continue undetected due to the nonspecific symptoms. Also, the incubation period of both viruses is 3–6 days.^1^ Such commonalities between the two viruses have led to delayed diagnosis and consequently postponed treatment and increased hospitalization. On the other hand, the major difference between RVFV and SARS‐CoV‐2 is the route of transmission: while the latter is transmitted through respiratory droplets, there have not been any confirmed cases of person‐to‐person transmission of RVFV^4^ (Table 1).

**TABLE 1:** Comparison between SARS‐CoV‐2 and RVFV

	SARS‐CoV‐2	RVFV
Common symptoms	Flu‐like febrile disease	Flu‐like febrile disease
Incubation period	3–6 days	3–6 days
Method of transmission	Through respiratory droplets	No person‐to‐person transmission

*Note*: These common traits make the differentiation between the two viruses challenging.

To date, there is no approved human vaccine against RVFV, although the virus is listed as a priority pathogen by the WHO because of its high potential to cause an outbreak and lack of effective precautionary measures.^1^ The preoccupation of healthcare workers in fighting COVID‐19 is slowing down research on a vaccine against RVFV, deeming the current RVF situation more encumbering. Moreover, some African countries have faced other infectious diseases and viral outbreaks such as bird flu, malaria, Ebola, measles, dengue, plague, Lassa fever, African swine fever, cholera, and HIV/AIDS.^11^, ^12^, ^13^, ^14^, ^15^, ^16^, ^17^, ^18^, ^19^, ^20^, ^21^

## 4 EFFORTS AND RESPONSES TO RIFT VALLEY FEVER IN AFRICA DURING COVID‐19 PANDEMIC

RVFV affects mostly livestock and is spread by mosquitoes. In particular, the Aedes mosquito is the main carrier and source of RVF outbreaks, and humans get infected directly via mosquito bite or through contact with infected livestock such as cows, sheep, camels, and goats.^4^

RVF is endemic in Africa, especially in areas where livestock is reared. Several control measures are implemented when an outbreak is declared: closing livestock markets and butcheries, imposing controls on movement, and banning raw milk.^22^

The impact of COVID‐19 on the economy and coexisting RVF outbreaks have presented a drastic challenge to both the economy and the fragile healthcare system of African nations.^23^ The difficulties of concurrently addressing COVID‐19 and RVF in Africa are associated with certain shortcomings such as insufficient resources and technical capacity, thereby impeding efforts against an appropriate response. Also, the lack of protective equipment, which when available is assigned to COVID‐19 relief efforts, has resulted in morbidity and mortality, as evidenced in some occupational groups.^24^

There is no known treatment for RVF, but it may be managed and prevented with the distribution and administration of vaccines. However, the supply for RVFV livestock immunizations has been insufficient and cannot protect populations on a large scale.^6^ As such, immediate actions need to be taken, such as increasing surveillance and educating the public on the health risks of RVFV infection, to promote the safety of Africa's civilians.

## 5 FUTURE RECOMMENDATIONS

The COVID‐19 pandemic serves as a pivotal moment that provides an unprecedented awareness of infectious diseases, highlighting essential yet unmet needs as well as multiple challenges in global healthcare and countries' health systems. For instance, the lack of research and development of evidence‐based practices in public health, universal health coverage, accessibility, awareness, and trustworthy information for the public have all contributed to healthcare limitations from a global standpoint. As a result, the increased risk of highly infectious disease outbreaks, such as RVF during the COVID‐19 pandemic, is significant and expresses a concern among specialists.^25^

RVF is a relevant disease affecting public health in the Arabian Peninsula, sub‐Saharan Africa, Egypt, and Madagascar, yet Asian countries have recently shown worrying signs of increased cases and transmission of zoonotic infections.^26^ For example, in the last few years, Korea has observed the presence of seroprevalence in local animals with no human cases yet to be registered, and China was the first to report human cases with infection related to RVFV imported from Angola.^2^, ^27^

The spread of RVFV to new countries alongside recurrent outbreaks in Africa and the overlapping COVID‐19 pandemic has set alarm bells ringing for a hazardous rise in disease transmission and possible damages to global health and economy.^26^

Because of RVF's regional distribution in the past, very few studies have been conducted regarding its manifestations, specificities, infection ratio, and potential illness in humans. As such, there is a crucial demand for evidence‐based data, with an emphasis on epidemiological studies and protocols in multiple regions as well as further studies on RVF in the context of the SARS‐CoV‐2 global pandemic.^1^, ^25^, ^26^

A One Health approach is highly favorable with regard to RVF response, as it underscores the development of a working group to explore and identify possible risk factors for infection and disease progression to severe presentation. Also, it is imperative to raise awareness about RVF among the governments and civil society, with health policy campaigns to increase education and subsequently prevent outbreaks.^1^

In this context, we recommend adopting an intersectional approach between different disciplines, including partnerships joining experts on animal and environmental health with local, national, and international healthcare workers in at‐risk regions.^1^, ^25^ An interdisciplinary approach is imperative to minimize damages to public health, society, and economy, to prevent infections in animals and humans, and to contribute to a sustainable recovery from the unprecedented global health crisis that is the COVID‐19 pandemic.^28^

## 6 CONCLUSION

RVF is one of the numerous diseases that disproportionately affect the lives of people in Africa. The recent rise in cases of RVF at an alarming rate has been attributed to the economic and healthcare consequences of the COVID‐19 pandemic. The WHO has designated RVFV as a priority pathogen due to its high epidemic potential and the lack of a licensed human vaccine or other effective countermeasures. Accordingly, RVFV requires urgent research and the development of new diagnostic tests, vaccines, and medications to prevent future outbreaks.

## FUNDING

We have not received any funding for this study.

## CONFLICT OF INTEREST

No conflict of interest declared.

## AUTHOR CONTRIBUTION

Olivier Uwishema: Conceptualization, project administration, Writing, reviewing, and designing

Tania Torbati: Review and editing the first draft

Helen Onyeaka: Reviewing and editing the second draft

Collection and assembly of data: All authors

Data analysis and interpretation: All authors

Manuscript writing: All authors

Final approval of manuscript: All authors

## Data Availability

Not applicable.
